# Cosmetics Preservation: A Review on Present Strategies

**DOI:** 10.3390/molecules23071571

**Published:** 2018-06-28

**Authors:** Noureddine Halla, Isabel P. Fernandes, Sandrina A. Heleno, Patrícia Costa, Zahia Boucherit-Otmani, Kebir Boucherit, Alírio E. Rodrigues, Isabel C. F. R. Ferreira, Maria Filomena Barreiro

**Affiliations:** 1 Antibiotics Antifungal Laboratory, Physical Chemistry, Synthesis and Biological Activity (LAPSAB), Department of Biology, Faculty of Sciences, University of Tlemcen, BP 119, 13000 Tlemcen, Algeria; noureddine.halla@univ-saida.dz (N.H.); z_boucherit@mail.univ-tlemcen.dz (Z.B.-O.); boucheritkebir@yahoo.fr (K.B.); 2 Laboratory of Biotoxicology, Pharmacognosy and Biological Recovery of Plants, Department of Biology, Faculty of Sciences, University of Moulay-Tahar, 20000 Saida, Algeria; 3 Centro de Investigação de Montanha (CIMO), Instituto Politécnico de Bragança, Campus de Santa Apolónia, 5300-253 Bragança, Portugal; ipmf@ipb.pt (I.P.F.); sheleno@ipb.pt (S.A.H.); 4 Laboratory of Separation and Reaction Engineering-Laboratory of Catalysis and Materials (LSRE-LCM), Polytechnic Institute of Bragança, Campus Santa Apolónia, 5301-253 Bragança, Portugal; 5 Laboratory of Separation and Reaction Engineering-Laboratory of Catalysis and Materials (LSRE-LCM), Faculdade de Engenharia, Universidade do Porto, Rua Dr. Roberto Frias s/n, 4200-465 Porto, Portugal; patriciasc@fe.up.pt (P.C.); arodrig@fe.up.pt (A.E.R.)

**Keywords:** cosmetic preservatives, microbiological safety, consumers’ protection, antimicrobial synthetic agents, toxic effects, preservatives efficacy

## Abstract

Cosmetics, like any product containing water and organic/inorganic compounds, require preservation against microbial contamination to guarantee consumer’s safety and to increase their shelf-life. The microbiological safety has as main goal of consumer protection against potentially pathogenic microorganisms, together with the product’s preservation resulting from biological and physicochemical deterioration. This is ensured by chemical, physical, or physicochemical strategies. The most common strategy is based on the application of antimicrobial agents, either by using synthetic or natural compounds, or even multifunctional ingredients. Current validation of a preservation system follow the application of good manufacturing practices (GMPs), the control of the raw material, and the verification of the preservative effect by suitable methodologies, including the challenge test. Among the preservatives described in the positive lists of regulations, there are parabens, isothiasolinone, organic acids, formaldehyde releasers, triclosan, and chlorhexidine. These chemical agents have different mechanisms of antimicrobial action, depending on their chemical structure and functional group’s reactivity. Preservatives act on several cell targets; however, they might present toxic effects to the consumer. Indeed, their use at high concentrations is more effective from the preservation viewpoint being, however, toxic for the consumer, whereas at low concentrations microbial resistance can develop.

## 1. Introduction

The global cosmetics market was $460 billion in 2014 and is expected to reach $675 billion by 2020 at an estimated growth rate of 6.4% per year [1]. This rising market requires continuous multidimensional control, namely, to monitor toxic ingredients and microbial contamination (i.e., chemical and biological contamination). Hazardous cosmetics pose a risk to consumers due to the presence of prohibited or restricted substances under the present in-force cosmetic laws. In addition, the contamination of cosmetic products is another risk for consumer’s health. According to the Rapid Alert System (RAPEX) of the European Commission (EC), 62 cosmetic products were recalled during the period between 2008 and 2014 due to contamination by microorganisms. The recalled products were notified by 14 different countries and their number was higher in 2013 and 2014 [2].

In general, the modification of cosmetic products is due to the presence of microorganisms, or might result from the exposure to atmospheric oxygen. To prevent these effects, two distinct groups of substances can be used, namely, antimicrobial preservatives, which act on microorganisms, and antioxidant preservatives capable of suppressing oxidation phenomena and the formation of free radicals [3]. In regulatory terms, a preservative is a substance of natural or synthetic origin intended to inhibit the development of microorganisms [4]. This inhibition should be effective over a broad activity spectrum and should have a duration longer than the cosmetic product itself, being equivalent to the expected shelf-life plus the usage time [5]. In addition, the antimicrobial activity should be sufficiently effective in order to prevent microorganism’s adaptation and resistance gain to the preservative system [6]. The cosmetic products are a nutrient-rich medium that favors microorganism’s growth, which, thereafter, influences the efficacy of the preservatives [3].

Considering the amount of antimicrobial agent to be used in a cosmetic, it is dependent on the intended role; high concentrations are used for active substances and low concentrations for preservatives. The first is used in antimicrobial cosmetics and the second one is required for most cosmetics. In addition to antimicrobial agents for preservation effects, the cosmetic industry applies other strategies, which include water activity and pH control, and the use of multifunctional ingredients.

In this context, this review discusses relevant available data concerning antimicrobial agents and cosmetic preservation. It has been divided into three sections; the first one is an overview of concepts with importance for the cosmetic field, microbiological safety, where a presentation of cosmetic products is given, in particular, those with antimicrobial properties. In addition, the contamination of cosmetics and the acceptance criteria of the different international regulations are detailed. The second section presents the various strategies used in the cosmetic preservation, together with the validation procedures required to introduce products on the market with microbiological safety. Considering that antimicrobial agents, particularly the synthetic ones, are the most used, the last section summarizes their importance and their application in cosmetic preservation. Herein the different chemical classes of these preservatives, toxicity, mechanisms of action as antimicrobials, and resistance mechanisms are discussed.

## 2. Overview of Cosmetics and Their Microbiological Safety

### 2.1. Definition and Classification of Cosmetics

The term ‘cosmetics’ derives from the Greek “Kosm tikos” meaning ‘having the power to arrange, skilled in decoration’, to give “kosmein”, to adorn, and “kosmos”, order, harmony [7]. The Council of European Union regulation gave the following definition: “cosmetic product means any substance or mixture intended to be placed in contact with the external parts of the human body (epidermis, hair system, nails, lips, and external genital organs) or with the teeth and the mucous membranes of the oral cavity with a view exclusively or mainly to cleaning them, perfuming them, changing their appearance, protecting them, keeping them in good condition, or correcting body odours” [4].

Generally, a cosmetic product is used in the direct treatment of the external surface of the human body in order to perform the following four functions: (1) maintenance in good condition; (2) change in appearance; (3) protection; and (4) correction of body odor [8,9]. The term “cosmeceutics” (or active cosmetics) was popularized by the dermatologist Albert Kligman in the 1980s. This term means a combination of cosmetics and pharmaceuticals, used to define products that can have a beneficial effect on skin, but cannot be considered as having a clear biological therapeutic effect (e.g., retinol, certain bleaching agents, etc.). However, the cosmeceutic term remains controversial without legal status and has not been generally accepted by all researchers [9]. Cosmetics can be classified according to their use, fields of application, functions, form of preparation, consumer’s age or gender, among others [10]. The most appropriate classification is as follows [4,9,11]: (1) cosmetics for personal cleansing (soaps, deodorants, shampoos); (2) cosmetics for the skin, hair, and integument care (toothpastes, products for external intimate care); (3) cosmetics for embellishment (perfumes, lip colors); (4) protective cosmetics (solar products, anti-wrinkle products); (5) corrective cosmetics (beauty masks, hair dyes); (6) maintenance cosmetics (shaving cream, moisturizing creams); and (7) active cosmetics (fluoridated toothpastes, antiseptics).

### 2.2. Cosmetic Products with Antimicrobial Effect

Cosmetic products with antimicrobial effect can be described as preparations with the ability to provide consumer’s protection against the presence of antimicrobial compounds, having bactericidal effect. Products like mouthwashes, skin disinfectants or antibacterial soaps present this characteristic. Currently, the limit between drugs and cosmetic products with antimicrobial effect is increasingly indistinct. Sometimes the difference between a cosmetic product and a drug lies in the concentration of the active ingredient in the product (e.g., mouthwash). There is also an unclear distinction between the definition of cosmetic and dermatological treatment (e.g., acne treatment). As a result, some modern cosmetics are in an increasingly grey zone and can almost be defined as drugs or over-the-counter (OTC). This fact confers a heavy responsibility on the various international regulation agencies [9,12]. In all cases, a decision on product qualification must be made by the competent national authorities on a case-by-case basis, and taking into account all relevant factors, such as their appearance, the type of active ingredient, length of use, mode of action, and claims. A proposal for classification, based on the international regulations, is presented in Table 1.

### 2.3. Microbiological Safety of Cosmetic Products

Generally speaking, all products, including cosmetics, containing water and organic/inorganic compounds under appropriate physicochemical conditions, are exposed to microbial contamination. This justifies why these products require effective and adequate protection against microorganism proliferation [13,14]. An ideal preservation system (intrinsic or extrinsic) should protect the product from microbial degradation, both in its original closed packaging until use, and in an open container throughout its use [15,16]. In recent years, the safety record for personal care products has been excellent, resulting in a scarce occurrence of infections due to contaminated products [17]. Studies have shown that the mostly frequent microorganisms found in cosmetics comprise *Pseudomonas aeruginosa*, *Klebsiella oxytoca*, *Burkholderia cepacia*, *Staphylococcus aureus*, *Escherichia coli*, *Candida albicans*, *Enterobacter gergoviae*, and *Serratia marcescens*, but also other bacteria, fungi and yeasts. The skin and mucous membranes are protected against microorganisms; however, their presence in these products can increase the risk of microbial infection [18].

**Figure 1:**
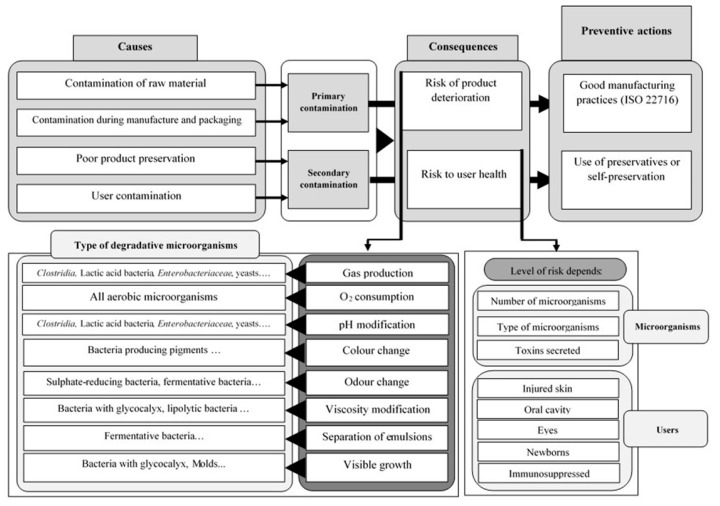
Causes, consequences. and ways of preventing cosmetics contamination [10,16,17,28,29,30,31,32].

Microbial contamination may occur during manufacture (primary contamination) and/or during consumer use (secondary contamination) [10,19]. The diagram in Figure 1 summarizes the causes, consequences, and ways of prevention against both types of contamination (primary and secondary). Moreover, all potential sources of contamination must be identified and monitored. In order to do so, four steps must be considered: (1) inspection and control of raw materials; (2) manufacturing process; (3) delivery of the final product and; finally; (4) its use by the consumer.

### 2.4. Microbiological Specifications According to International Regulations

With industrialization and the fast emergence of new ingredients used in cosmetics, several directives and regulations have been elaborated, in order to control the use of these ingredients, to ensure consumer safety, to determine the responsibilities, and enable claims for adverse reactions. Among the recommended regulations worldwide, only three represent the major cosmetic markets, namely the United States, the European Union, and Japan [33].

#### 2.4.1. Legislation in the United States

In the United States, the FDA (U.S. Food and Drug Administration) is the lead agency for the enforcement of laws governing the marketing of cosmetics. It is responsible for controlling cosmetic products after they are placed in the market [34,35].

The FDA prohibits the distribution of adulterated or mislabeled cosmetics. In addition, FDA has banned the production of cosmetic products under conditions that could lead to contamination. Although it is not mandatory, cosmetics must be manufactured in accordance with current good manufacturing practices (CGMPs). The FDA declares that cosmetics should not be sterile, however, they should not be contaminated with pathogenic microorganisms and the density of non-pathogenic organisms should be low [36].

Since the FDA does not specify acceptable levels, the cosmetic industry generally follows the guidelines of the Personal Care Products Council (PCPC) (formerly the Cosmetic, Toiletry, and Fragrance Association (CTFA)) regarding the level of microbial contamination and the absence of pathogens: (1) for the eye zone and products for babies, it should not be greater than 500 colony forming units (CFU)/g; (2) for all other products, it has to be no greater than 1000 CFU/g [37].

#### 2.4.2. Legislation in Japan

In Japan, cosmetics are regulated by the Ministry of Health, Labor, and Welfare (MHLW) under the Pharmaceutical Affairs Law (PAL). For legal reasons, cosmetics are divided into quasi-drugs and cosmetics. The Japanese Pharmacopoeia (PJ) was established and published to regulate the properties and qualities of medicines by MHLW on the basis of the provisions of Article 41 (1) of the Act, following advice from the Pharmaceutical Affairs and Food Sanitation Council (PAFSC). Since it was first published in June 1886, the PJ has been revised several times. The last PJ edition (17th edition) was published in 2016. The Japanese Pharmacopoeia harmonized the criteria for accepting the microbiological quality of non-sterile pharmaceuticals [38].

Microbiological quality acceptance criteria require that the total number of aerobic microorganisms in products for oromucosal, gingival, cutaneous, and nasal uses, should not be greater than 10^2^ CFU/g or CFU/mL and a total combined number of yeasts/molds should not be greater than 10^1^ CFU/g or CFU/mL in the absence of *Staphylococcus aureus* and *Pseudomonas aeruginosa* in 1 g or 1 mL of the product [38].

#### 2.4.3. Legislation in the European Union

In the European Union (EU), cosmetic products have been regulated by EU Council Directive 76/768/EEC. These rules were adopted on 27 July 1976 and at 27 September 1976 were published in the Official Journal of the European Communities “L 262”. Since then, it has been constantly evolving and adapted to technical progress [4].

Recommendations on the limits of microbial contamination in cosmetic products can be found in the SCCS ‘Scientific Committee on Consumer Safety’ Guideline “SCCS Notes of Guidance for the Testing of Cosmetic Ingredients and their Safety Evaluation, 9th revision”. Two distinct categories of cosmetic products are defined within the limits of microbiological quality control:

- Category 1—products specifically intended for children under three years, to be used in the eye area and on mucous membranes;
- Category 2—other products.

It is generally accepted that for cosmetics classified in Category 1, the total viable count for aerobic mesophyllic microorganisms should not exceed 10^2^ CFU/g or 10^2^ CFU/mL of the product. For cosmetics classified in Category 2, the total viable count for aerobic mesophyllic microorganisms should not exceed 10^3^ CFU/g or 10^3^ CFU/mL of the product. *Pseudomonas aeruginosa*, *Staphylococcus aureus*, and *Candida albicans* are considered the main potential pathogens in cosmetic products. These specific potential pathogens must not be detectable in 1 g or 1 mL of a cosmetic product of Category 1 and in 0.1 g or 0.1 mL of a cosmetic product of Category 2 [18].

In 2015, a new standard was published by the International Organization for Standardization (ISO 17516:2014 Cosmetics-Microbiology-Microbiological limits), in which the main objective is to define acceptable quantitative and qualitative limits for finished cosmetic products. This standard requires that each manufacturer be responsible for the microbiological safety and quality of its products and must ensure that they have been produced under hygienic conditions. Cosmetics are not supposed to be sterile. However, they must not contain excessive quantities of specified microorganisms or microorganisms which may affect the quality of the product or the safety of the consumer [39].

## 3. Preservation Strategies

Manufacturers of cosmetics use different strategies to prevent microbial contamination without affecting the properties of the product itself. Usually, the term preservation refers to the use of synthetic and natural chemical preservatives. However, self-preservation or free preservation is a preservation without the use of an additional chemical ingredient classified as preservative in the annexes of the cosmetic legislation [40,41]. The microbial preservation strategies range from the first stages of manufacture to consumption, in order to minimize the risk of microbial contamination. The main stages of this procedure will be briefly described. In addition, all the strategies mentioned below, with the exception of the synthetic chemical preservatives used, are introduced by several authors in the concept of “Hurdle Technology” for the preservation of cosmetics. ‘Hurdle Technology’ is a term that describes the intelligent combination of the several factors that prevent the development of microorganisms [42,43,44,45,46].

To achieve a good protection of cosmetic products against microbial contamination, the industry provides two stages of preservation: primary and secondary. The strategy of primary preservation occurs during manufacturing and is based on the application of GMP. The secondary preservation, which takes place after manufacture, uses chemical, physical, or physicochemical ways to attain an efficient protection.

### 3.1. Primary Preservation Strategy

GMP must be strictly obeyed during the production of cosmetic products. The preparation of the cosmetics under strictly aseptic conditions must avoid their microbial contamination. Water treatment, microbial control of raw materials, equipment disinfection, and qualification of personnel can reduce the risk of contamination [10,30,42].

Certification, ISO 22716:2007—Good Manufacturing Practices (GMP) for Cosmetics, has been approved and accepted (with or without modification) by most regulatory organizations around the world, particularly after the July 2008 meeting of the International Cooperation on Cosmetic Regulation (ICCR) (the United States, the European Union, Japan, and Canada) [47].

### 3.2. Secondary Preservation Strategy

Three main strategies have been used to preserve cosmetic products during storage, transport, and use: physical, chemical, and physicochemical preservation.

#### 3.2.1. Physical Secondary Preservation

This type of preservation is completed by the use of primary packaging where a physical barrier exists to prevent microbial contamination. Two levels of protection can be provided by the packaging: (1) against contamination during use; and (2) against accumulation of contamination in the distribution system [48]. The shape and characteristics of primary packaging presents a significant influence in the potential for microbial contamination. These characteristics include not only the physical configuration of the packaging (boxes, jars, bottles, flasks, sachets, tubes, aerosol propellants, etc.), but also the nature and composition of the used materials (polymers, glass, etc.) [30,32,42,49]. For example, jars and bottles are more likely to cause microbial contamination, whereas closed system configurations (with airless pumps) are less accessible to contamination [50]. Compressed gases, as aerosol propellants, generally provide a good protection to the product. The pumping systems and tubes containing narrow openings also represent an excellent design for product protection during use. Moreover, the risk of contaminated bath water from shampoos and shower gels during use is greatly diminished by the use of containers with a narrow opening [20,51]. Additionally, the use of re-closable systems can reduce the potential for microbial risk. Beyond this, the sizes of the packaging and the delivery holes may also have an effect on exposure and microbial risks. However, the primary packaging system can influence the effectiveness of chemical preservatives by migration or adsorption phenomena [49,52]. In the last decade, active packaging technology (packaging incorporated with antimicrobial agents) has been transferred from food to the cosmetic field [53].

#### 3.2.2. Physicochemical Secondary Preservation

##### Water Activity

Usually, water is the major constituent of cosmetics, but it is an ideal growth factor for microorganisms. To solve this problem, certain substances can reduce the water activity (aw), such as salts, polyols (sorbitol, glycerol, ethoxydiglycol, etc.), protein hydrolysates, amino acids, and hydrocolloids (xanthan gum, guar gum, etc.), glyceryl polyacrylate gel, sodium polyacrylate and sodium chloride. The choice of these substances depends on their aspect, their toxic effect, and also the nature of the cosmetics [30,32,42,54,55]. Water activity can also be reduced by the use of vapour-resistant bottles, film strip, vapour-repellent film coatings, or polyacrylamide hydrogels [56]. Berthele et al. [57] reported that a water activity value of 0.8, and without preservatives incorporated in the formulas tested, can guarantee microbiological stability of the cosmetic products.

##### Emulsion Form

Water-in-oil (W/O) emulsions can minimize the risk of microbial contamination more than oil-in-water (O/W) emulsions [42]. The size of the emulsions droplets can improve the cosmetics effectiveness. In many cases, the decrease in the size of the emulsion droplets (nanoemulsion) increases the antimicrobial activity. However, the antimicrobial activity depends also of the oil phase chemical composition, namely the type of phenolic compounds, their concentration, and chemical structure [58,59,60].

##### pH Control

The optimum pH for microorganism’s growth in cosmetic products is between 5 to 8, meaning that any pH outside this range induces unfavourable conditions, thus decreasing their growth rate [6,42]. The acidic pH of cationic hair conditioners (pH = 4, approximately) contributes to the antimicrobial action of these products [54,61]. Other formulations with acidic pH can inhibit the growth of microorganisms, such as products containing salicylic acid and aluminium compounds in antiperspirants (pH ranging from 3.5 to 4.5) [62]. Liquid soaps having an alkaline pH (pH 9.5 to 10.5) exhibit an unfavourable environment for microorganism growth of (e.g., destabilizing their membrane), due to the effects of ionized fatty acids and free alkalinity of the existent NaOH. Generally speaking, microorganisms cannot proliferate or survive in a cosmetic formulation with a pH of less than 4 or greater than 10 [54,57].

#### 3.2.3. Chemical Secondary Preservation

##### Synthetic Chemical Preservatives

The EU Cosmetic Directive means by preservative substances that are exclusively or mainly intended to inhibit the development of microorganisms in the cosmetic products. Their presence is essential in most cosmetic products. The choice of these preservatives as ingredients in cosmetics must comply with Annex V of the cosmetic regulation (Article 14 of the Cosmetic Regulation) [4]. Generally, preservative selection is based on three criteria (plus the regulatory criterion): (1) very good antimicrobial efficacy; (2) non-toxic; and (3) compatible with the other ingredients of the cosmetic formulation [63,64]. Currently, preservatives have been used as a mixture to increase antimicrobial activity, broadening the spectrum of activity, reducing the resistance of microorganisms and the risk of toxicity [65].

##### Natural Chemical Preservatives

Plant extracts and essential oils are mainly added to cosmetic preparations due to their well-recognized properties, such as: antioxidant anti-inflammatory and antimicrobial, emollients, dyes, humectants, wound healing, anti-mutagens, anti-aging, protective agents against UV-B damage, and reducing skin discoloration [66]. Several studies have shown the preservative efficacy of natural products in cosmetic products [29,67,68,69,70,71,72,73,74,75,76,77,78]. Natural products are used free, microencapsulated, or transported by nanostructured carriers [79,80]. Their application as antimicrobials in cosmetic preparations is often discouraged due to their loss of activity in dilutions, pH-dependency, volatility and lipophilic aspects (essential oils), and strong odor (essential oils), which can be highly inadequate/undesirable for some kind of products [6,30,32,42,81].

##### Multifunctional Ingredients

Each ingredient is added to the cosmetic formulation for a well-defined function, but it can, simultaneously, contribute to another effect (such as antimicrobial activity), thus acting as a multifunctional ingredient. In the sense of self-preservation, these ingredients have been used as antimicrobial preservatives by replacing conventional preservatives. Chelating agents, surfactants, humectants, and phenolic compounds are examples of multifunctional ingredients. Chelating agents (e.g., EDTA ‘ethylenediaminetetraacetic acid’, GLDA ‘glutamic acid, *N*,*N*-diacetic acid, lactic acid, citric acid, and phytic acid) increase the permeability of cell membranes and make them more sensitive to antimicrobial agents. In addition, chelating agents block the iron required for metabolism and microbial growth, and can enhance the antimicrobial efficacy of the used preservatives [42,82]. Surfactants with antimicrobial properties are the 1,2-diols (from butanediol to octanediol, mainly caprylyl glycol) due to their amphiphilic character and average molecular size, exhibit viscosity modulation properties that complement their antimicrobial properties. These properties depend on the length of the chain and the position of the hydroxyl groups [83,84]. Medium-chain saturated fatty acids, such as heptanoic acid (C7), caprylic acid (C8), capric acid (C10), and lauric acid (C12), and their esters with glycerine or propylene glycol, have been found to be active against enveloped viruses and various bacteria and fungi. In the case of glyceryl monoesters, there is an emulsifier passage to the antibacterial activity at the C8 to C12 ranges [42,85]. Other ingredients, such as phenethyl alcohol and cationic detergents, are used as emulsifiers, and have intrinsic antibacterial properties [6,42]. The use of humectants, such as glycerin, sorbitol, and xylitol, at sufficient levels, increases the strength of the formula [6]. Amaral et al. [86] reported that monoester c-8 xylitol can be used as an alternative preservative for cosmetic formulations. In a dental cream, a mixture of sorbitol and glycerin, at 10% to 12% levels, is often enough to protect the formula [6]. Berthele et al. [57] observed that a high concentration of glycerin, beyond having an influence on the appearance of the product, it could also present an effect on the microbial growth. The primary function of phenolic antioxidants is to delay the self-oxidation of unsaturated oils that could influence the color and odor of the product. Beyond that, compounds as propylic gallate, caffeic acid, coumaric acid, ferulic acid, citric acid, and tartaric acid have also demonstrated antimicrobial activity [87].

### 3.3. Validation of Effective Preservation

A proper preservation ensures effective protection against the undesirable growth of microorganisms during storage and product use. To meet these requirements, the choice of the type and concentration of preservative during formulation development is important, but, likewise, the type and extent of potential microbial influences that could impair the quality of the final product should be considered. The microbial quality of raw materials is a particularly important factor, but the provision of complete production instructions, covering the treatment of preservatives and the hygiene of raw materials until the final product is shipped, is also vital [88]. We have cited above the different strategies of preservation, but before delivering the final product, most cosmetic manufacturers will ensure three important steps in order to preserve the product, namely: (1) choice of primary packaging; (2) microbiological control of the raw material; and (3) validation of the antimicrobial efficacy of the preservation system.

#### 3.3.1. Types of Primary Packaging

The type of primary packaging also affects the protection of the product in use by the consumer (see Section 3.1). Packaging can pose a microbial hazard before filling it with the ingredients of the cosmetic product. Today, cosmetics wrapped in wide-open bottles are one of the biggest challenges for any preservation system, with their large surface area exposed to a damp, contaminated environment [89].

#### 3.3.2. Microbiological Control of Raw Materials

During manufacture, the main sources of contamination are the used raw materials, including water, and the manufacturing process itself. The microbiological quality of water depends on its origin. Water remains one of the most important factors in the contamination of a product. Species such as *Pseudomonas*, *Achromobacter*, *Aeromonas*, *Flavobacterium*, *Xanthomonas*, *Actinobacter*, and *Aerobacter* spp. were recovered from natural waters. The presence of *Escherichia coli* may be a sign of recent contamination by wastewater [2,90]. Treatments by softening or deionizing water often change the microbiological quality of the water. These microbiologically-treated water systems must be well maintained using, for example, ultraviolet (UV) and/or bacterial filtration to ensure optimum quality [91,92]. The raw material of animal or vegetable origin can be highly contaminated by coliforms [93]. However, the synthetic raw materials are relatively free of contamination, with the exception of some that have additional stages in their manufacture, such as kaolin, sugars and vitamins, synthetic surfactants, or hydrated salts [92].

During the manufacturing process, contamination can occur by contact with operators, manufacturing equipment, and air. Microorganisms from human sources are likely to contaminate a cosmetic product; they can be part of the nasopharynx, the oral flora, the hair, the skin of the hands and, under certain circumstances, the intestinal flora. Among these, fecal *streptococci*, *staphylococci*, *enterobacteria*, and *Pseudomonas* have enough vitality to survive, and even to multiply, within a product [94]. Manufacturing equipment is also an important source of contamination, from maintenance materials (oils, greases), poor cleaning and/or disinfection on a regular basis, and product change. The cleaning-in-place (CIP) design must be carefully evaluated [95]. Particular attention must be paid to air quality of the manufacturing chambers. The number of workers together with the size of their movements, contribute to 80% to air contamination [96]. Air conditioning contributes to 15% of this contamination, and the chamber structure (materials used on its construction) contributes to 5%. It is, therefore, essential to set acceptable levels for biocontamination of air and to its quality control [92].

#### 3.3.3. Antimicrobial Efficacy Test of the Preservation System

The antimicrobial efficacy test is used to assess the efficacy of preservation systems in the final product. The antimicrobial efficacy test was initially designed to assess the performance of antimicrobials added to inhibit the growth of microorganisms that may be introduced into the product during or after the manufacturing process [97]. Several tests have been recommended by different laboratories, but the challenge test (described next) remains the method adopted by the international regulations. These methods are described in the European, American, and Japanese pharmacopoeia, as well as other organizations, such as PCPC (Personal Care Products Council) (from CTFA-M1 to CTFA-M7), ASEAN (Association for Southeast Asian Nations), ASTM (American Society for Testing and Materials), and International Organization for Standardization (ISO 11930 standard), among others.

##### Challenge Test

The challenge test is used during product development to determine the efficacy and stability of the preservative system over time. The test involves inoculating a measured amount of product with known amounts of microorganisms (bacteria, yeasts, and molds) [98]. Whenever possible the original packaging is used for the test. The containers are protected from light and incubated at room temperature for 28 days. The mortality rate is measured over this period in relation to the acceptance criteria set out in the official regulations documents [97,99].

**Figure 2:**
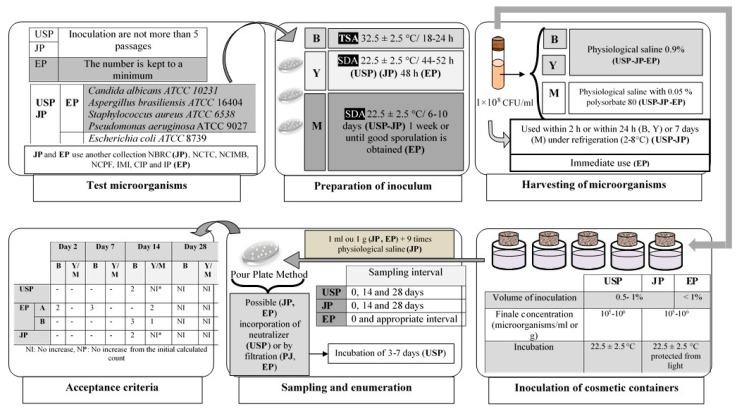
Preservative effectiveness testing comparison between the Japan, USA and European Pharmacopeias [38,108,109] where: B: bacteria, Y: yeast, M: molds, USP: United States pharmacopeia, JP: Japanese pharmacopoeia, EP: European pharmacopoeia, TSA: soybean-casein digest agar, and SDA: sabouraud dextrose agar.

Challenge test assessment is related to the stability of a formulation during manufacture, storage, and its use by the consumer. It is recommended that all these aspects be duly taken into account when performing such tests by carrying out the following parameters: (1) validation of the preservation efficacy when freshly prepared in laboratorial conditions; (2) validation of the preservation efficacy after the end of storage in the container, to show possible interference with the packaging materials; and (3) validation of preservation efficacy in the first production batch, just prior to packaging, thus revealing all possible influences occurring throughout the manufacturing process [100]. To evaluate the microbiological quality of a product, results of the efficacy test of a cosmetic product preservatives are collected and a prognosis is achieved [99]. The recommendations of the challenge test are inspired by the European, American, and Japanese pharmacopoeia. A comparison between these three pharmacopoeias is summarized in Figure 2.

a. Test organisms

The specific strains recommended to be used in these tests can be obtained from official cell culture collections, such as the American Type Culture Collection (ATCC). The most common test strains are potentially pathogenic representatives of Gram-positive bacteria (*Staphylococcus aureus*), Gram-negative bacteria (*Escherichia coli* and *Pseudomonas aeruginosa*), molds (*Aspergillus niger*), and yeast (*Candida albicans*) [17].

*Staphylococcus aureus* represents Gram-positive cocci in many tests. It is a part of normal nasal and cutaneous microflora. Although rare, its presence in cosmetic products may be indicative of human contamination. *Pseudomonas aeruginosa* is a Gram-negative bacilli. It is a well-known and highly pathogenic ubiquitous bacteria. It also shows high resistance against many preservatives. *Escherichia coli* is a Gram-negative bacilli of the family Enterobacteriaceae. It is considered as an indicator of fecal contamination. Like most coliform bacteria, it can easily develop resistance to preservatives. *Candida albicans* is present in human mucous and ubiquitous in the environment. It is the representative of yeasts being an example of yeast resistance to presents to preserved systems. *Aspergillus niger* is a major cause of product decomposition and contamination by filamentous fungi [6,17,101].

The conservation of strains is an important factor. For example, most bacteria and the yeast *Candida* remain viable for one month under refrigerated conditions, while *Pseudomonas aeruginosa* cannot be useful after two weeks (depending on specific conditions). An effective way to keep mold spores is to store them at room temperature on slanted agar. Weekly or periodic transplanting may be done to ensure the viability of microorganisms, but this practice increases the risk of resistance loss. Alternatively, the cultures can also be frozen or lyophilized, in order to maintain the stability of the microorganism and avoid the need of frequent subcultures. The main advantage of these storage media is the prevention of genetic resistance factors loss [28,102].

b. Inoculum

Strain maintenance is an important component of any standard protocol, and involves standardization of strain storage, culture conditions (time and temperature), and selected nutrient medium [103]. The growth and preparation of a test organism determines its physiological state and have a direct influence on the results of the preservative efficacy analysis [104,105]. It is essential to maintain cultures of microorganisms that are transplanted on suitable supports, to ensure viability and resistance [103].

A medium such as tryptic soy agar (soybean-casein digest agar) supports vigorous growth and is recommended for the initial culture of the bacteria. Sabouraud dextrose agar is a non-selective medium used for the cultivation and conservation of pathogenic and non-pathogenic fungi [106].

Pharmacopoeia use saline solutions to wash test strains before inoculation instead of nutrient broth. The latter decreases the inactivation rate of the test organisms comparatively with the saline solution prepared for the strains grown on the agar [107].

According to all the three pharmacopoeia, the strains are cultured for the same period of time, ensuring that the cells are viable and growing in the log phase, thereby normalizing the response to antimicrobial agents [38,108,109].

c. Inoculation of samples

After adjusting the number of starting cells, the inoculum is then used to inoculate test samples. For some organizations (such as CTFA), samples of cosmetic products can be inoculated as bacterial or fungal “cocktails”. Nevertheless, the use of bacterial or fungal mixtures offers considerable savings in time and cost. However, the three pharmacopoeias recommend inoculation by a single strain separately. The volume of the inoculum should not exceed 1% of the product sample, in order to avoid the modification of its physical and chemical properties [38,108,109].

The inoculated test samples are incubated during 28 days, varying the conditions between room and high temperature, depending on the objective, since higher temperatures are used to simulate specific environmental conditions. Temperatures between 20–25 °C support the growth of microorganisms and their possible reaction with preservative active ingredients [98].

d. Assessment of the microbial level for cosmetic products

To estimate the level of microorganisms inoculated in a sample of a cosmetic product, it is required to select the appropriate conditions of each culture (culture medium, dilution, temperature and period of incubation). These conditions must provide an unlimited growth of microorganisms, resulting in the inactivation of the preservative system present in the sample [102].

The number of viable microorganisms’ existent in the inoculums suspension is determined by the plate count method, through which the initial concentration of CFU/mL in the test product is determined. The inoculated vessels are examined 7, 14, 21, and 28 days after inoculation and the number of microorganisms (CFU/mL) is determined at each time interval, being the percentage of microorganisms estimated relative to the initial concentration [28].

The preservative inactivation is considered successful when the number of the microorganisms inoculated at zero time deviates by no more than 1 log10 from the one theoretically predicted. The survival rate can be either qualitatively or quantitatively evaluated [110]. Several independent researchers have applied other microorganism counting methods in the efficacy test of preservatives, including impedance, direct epifluorescence (DEF), and ATP bioluminescence (ATP-B).

The impedance method is based on a calibration between CFU and the impedance detection time (DT) establishment. In this method, the electrochemical changes in a microbiological culture due to microorganisms’ metabolism is measured [111]. In a culture medium, the impedance variation occurs due to the chemical composition modification caused by the growth of microorganisms and metabolic activity. The density of the population of microorganisms is correlated with the DT of the impedance. The DT is referred to as the time required to produce a detectable acceleration in the impedance curve [112]. The results obtained indicated that this method is applicable to the entire range of test strains (bacterial and fungal), having a detection sensitivity equivalent colony counting method, representing a satisfactory alternative to this one [113,114]. In 2014, Ferreira et al. [115] used lyophilized inoculum of solid powders in order to enable the microorganisms’ homogenization in the sample. They also verified the applicability of the impedance method for these lyophilized inoculum.

The direct epifluorescence (DEF) method is based on the observation that viable microbial cells, which mainly contain RNA, are stained in red with orange acridine, while non-viable cells, which mainly contain DNA, are stained in green. The DEF, as a quick method, has two major advantages: first, it gives an immediate result (between 1 to 4 h); and second, it presents the potential for high detection sensitivity which is determined by the maximum sample volume that can be concentrated on the filter. However, in practice, there are problems associated with the interference of cellular debris with viable cells (red stain), as well as interference of dead clumped cells with microcolonies (green fluorescence). The clumping of bacterial cells by some preservatives (chlorhexidine) is another problem which overestimates the viability. Thus, this technique is not applicable to *Aspergillus* and it is not suitable for processing complex formulations that cause problems in filtration of samples [116].

In the ATP bioluminescence method (ATP-B), the bioluminescence mechanism involves the enzyme luciferase in the presence of luciferin, oxygen (O_2_), magnesium and ATP. This reaction leads to the emission of photons and the intensity of the light produced is directly proportional to the rate of ATP [117]. However, this method is not applicable to the genus *Aspergillus*, and to creams or suspensions, since these latest could interfere with the detection of light emission [116].

e. Interpretation of results

The acceptance criteria, in terms of the logarithmic reduction of the viable microorganism’s number relatively to the value obtained for the inoculums, vary for the different categories of preparations, according to the international organizations [118]. The criteria of the three pharmacopoeias for the evaluation of antimicrobial activity are given in Figure 2. The log reduction is calculated by the following equation: log reduction = log of initial CFU/mL-log of product challenge results CFU/mL [98].

##### Other Published Methods

Preservatives should have a rapid effect against a wide range of microorganisms. Several screening methods and the assessment of preservation effectiveness have been reported as the D-value method and the capacity test, both described below.

a. D-value method

In 1979, Orth proposed a quick method to estimate the effectiveness of preservatives [119]. This method can be used to determine the shelf life of cosmetic products within 48 h for bacteria and yeasts, and seven days for mold. The inactivation rate of the selected organisms is given by the decimal reduction time (D-value). The D-value, for each organism in each test sample, is calculated by taking the negative inverse of the slope of the straight line obtained by linear regression of the logarithm curve of surviving organisms, after the inoculation in the tested sample. To determine the D-value, the following conditions must be fulfilled: (1) one strain for each test; (2) a quantitative determination of the number of viable microorganisms; (3) preservation must reduce the number of microorganisms by several orders of magnitude, within the first 24 h; (4) the death curve must adjust a linear regression; and (5) sufficient data are acquired at the first reading point to generate the regression [120].

b. Capacity test

The capacity test evaluates the effectiveness of the preservative concentration and, thus, the spectrum of antimicrobial activity of the creams, suspensions, and solutions. This test involves the use of mixed bacterial and fungal cultures (yeasts and molds). A sample with a mass of 20 g is inoculated with 1 mL of the mixed culture. After 48 h of incubation at room temperature, 1 mL of each sample is removed and re-seeded in broth added with a suitable neutralizer. A sample of this dispersion is then spread on the neutralizer-containing agar. A preservative should reduce the number of viable organisms in a 103 inoculated formulation, within 48 h, for creams and suspensions, to produce a single negative result. This capacity decreases gradually due to the dilution and absorption of preservative by the microorganisms. After each test, the products are sampled and challenged again until the product receives 15 challenges without showing growth (a well-preserved product) or until three consecutive positive results occur (a less-preserved product) [28].

##### Factors affecting preservation effectiveness tests

The effectiveness of a preservation system can be affected by the quality of the raw materials and several other factors with influence in the microbiological quality of a complete formulation [121].

a. Preparation of the inoculums

Considering the inoculums preparation, Muth suggested that there is no difference between freshly-prepared inoculums and a frozen preparation [28]. The use of solid culture media limits the growth of colonies and adherent biofilms. Moreover, it can also confer properties to the cells that are not expressed in liquid media. In addition, some studies have also pointed out that low molecular weight agar-agar-derived polysaccharide materials can be taken at the same time as cells [104,122]. The size of the inoculums may also have an effect on the apparent activity of the antimicrobial agent. The inoculum must have an appropriated size, enough for allowing the reduction evaluation [123].

b. Adjustment of the inoculum

The cell density will affect several of the biological properties of bacterial suspensions during tests of antimicrobial activity. In order to normalize the cell densities in the inoculum, it is often needed to, first, concentrate the cells and, after, dilute them in solutions until the desired concentration is reached. When cultures are in liquid medium, the concentration of the cells is achieved either applying centrifugation or by membrane filtration. The conditions used during centrifugation subject cells to high hydrostatic forces that can provoke damage at the cellular level. For some species, a significant proportion of the initial cell population is killed by centrifugation, especially when they are collected in the logarithmic growth phase [122].

c. Cell harvesting

In his work, Orth detected a decrease in the antimicrobial activity of the inoculums prepared in a broth, comparatively to the one observed with a saline solution of cells cultured on agar [107]. The procedures of harvesting the cells can be extremely damaging. Thus, changes in the suspension medium, osmolarity, temperature, and pH at the same time have been reported as affecting the cell viability. Bacterial cells have a remarkable ability to adapt their phenotypes to the extremes of the physicochemical environment when the exposure is progressive, however, if the same conditions are suddenly imposed, the cells will not survive [122].

d. Used formulation

The chemical and biological activities of a preservative can be influenced by the overall formulation of the product. Surfactants, nonionic in particular, can influence the activity of preservatives, especially in oil-based emulsions. In addition, the buffer system and the water activity may also have effect on the preservative mode of action. The level of solids present in a formulation can also affect the effectiveness of a preservative [28]. The type of container used for conditioning a cosmetic product will influence the concentration and activity of a preservative [89].

e. Microbial count

The used culture media have a direct effect on the antimicrobial efficacy test of preservatives. It is well established that some media, while capable of sustaining the growth of normal microbial cells, are incapable of supporting the growth of stressed microorganisms [124]. In addition to the nutrient properties of culture media, the temperature and extent of incubation are important factors for the carrying on of microorganism proliferation [28]. Additionally, some authors recommend at least three repetitions of the plate counting. Errors in sampling, dilution, and the use of uncalibrated pipettes must be considered [125].

## 4. Synthetic Chemical Preservatives

This section will discuss the most used preservatives in cosmetics listed in Annex V of the Regulation (EC) No. 1223/2009. It is worth mentioning that, in the following section, the nitrogen compounds, formaldehyde releasers, isothiazolinones, and the quaternary ammonium compounds will be enclosed in different classes due to their specific properties. The nitrogen compounds used as preservatives according to Annex V of the EU Directive are: zinc pyrithione, triclocarban, piroctone olamine, chloroacetamide, hexamidine, dibromohexamidine isethionate, dimethyloxazolidine, climbazole, iodopropynyl butylcarbamate, 7-ethylbicyclooxazolidine, and ethyl lauroyl arginate hydrochloric acid [4].

Currently, the cosmetic industry suffers from a considerable lack of less-toxic preservatives, with regulations updating the limits of their use periodically. For this reason, there is considerable interest in finding effective and safe alternative preservatives. Future alternatives seek a broad spectrum against microorganisms with a better safety profile. Compounds with good antimicrobial properties and low toxicity, such as plant extracts, are interesting future alternatives. In addition, the development of preservative-free products is also of particular interest today.

### 4.1. Different Chemical Classes

**Figure 3:**
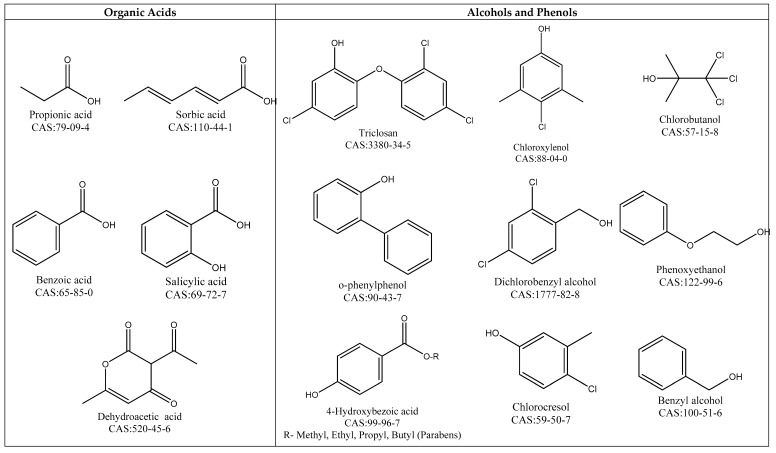

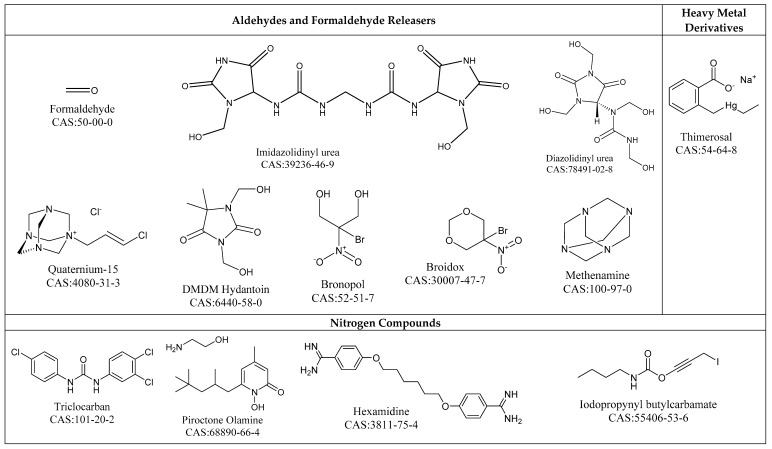

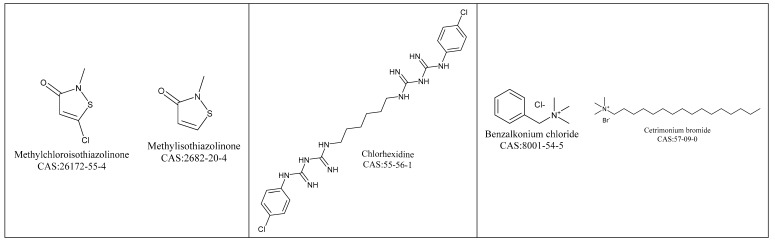
Chemical structures of some preservatives used in cosmetics.

The most commonly used antimicrobial preservatives are presented in Figure 3. These can be divided according to their chemical composition, namely: organic acids, alcohols, and phenols, aldehydes, and formaldehyde releasers, isothiazolinones, biguanides, quaternary ammonium compounds (QAC), nitrogen compounds, heavy metal derivatives, and inorganic compounds. Detailed information about the mechanism of action of these antimicrobial preservatives is given below.

#### 4.1.1. Organic Acids

The organic acids are active if the carbon number of the alkyl chains is high, decreasing, however, their solubility in water. pH is considered to be a major determinant of the organic acids’ effectiveness because it affects the concentration of formed undissociated acids [126]. Uncharged molecules are those forms that enable the penetration of organic acids into the cell, however, the antimicrobial efficacy of most organic acids is presented by their dissociated form [127]. The acidic pKa of these preservatives should be controlled since a pH change of 1.5 or more above the neutrality may cause the progressive loss of antimicrobial activity [128].

The most important organic acids referred in the Annex V are: benzoic acid, propionic acid, salicylic acid, sorbic acid, dehydroacetic acid, formic acid, undecylenic acid, citric acid, and sodium hydroxymethylaminoacetate [4]. In 2014, the European Commission added the mixture of citric acid and silver citrate to Annex V and allowed its use as a preservative up to a maximum concentration of 0.2% corresponding to 0.0024% of silver. It should not be used in oral and eye products [129].

#### 4.1.2. Alcohols and Phenols

From the chemical structure of the phenols, it has been observed that: (1) the para-substitutions of the alkyl chain with six carbon atoms increases their antibacterial activity. In addition, linear para-substituents provide higher activity than branched chain substituents containing the same number of carbon atoms [128]. On the other hand, Park et al. [130] reported that the activity does not depend on the length of the para-substituted phenol side chain; (2) the halogenation increases the antibacterial activity of the phenols. When the alkyl group is in the ortho position and the halogen is in the para position, the phenols will have greater antibacterial activity; (3) nitration has the advantage of increasing the activity with respect to bacteria by the modification of the oxidative phosphorylation; (4) in the bisphenol series, the activity is linked with the two C_6_H_5_ rings which are separated by -CH_2_-, -S-, or -O- groups. If the groups are -CO-, -SO-, or -CH (OH)-, the antimicrobial activity drops. Furthermore, it has been found that the halogenation of bisphenols and the presence of the hydroxyl groups in the 2,2′-position contribute to the antimicrobial activity of the bisphenols [128].

The preservatives of this class, which are included in the positive list, are: parabens, triclosan, chlorobutanol, o-phenylphenol, chlorocresol, chloroxylenol, phenoxypropanol, benzylhemiformal, phenoxyethanol, dichlorobenzyl alcohol, benzyl alcohol, o-cym-5-ol, chlorophene, chlorphenesin, and bromochlorophene [4].

In 2013, benzyl alcohol was included in Annex V [131]. Moreover, an amendment was published in the Official Journal on 9 April 2014, which also limits triclosan to a maximum concentration of 0.2% in mouthwashes and 0.3% in special cosmetic products, such as toothpaste, hand soaps, body soaps, and face powders [132]. In these amendments, five parabens were added to the prohibited substances list in cosmetic products described in Annex II: isopropylparaben, isobutylparaben, phenylparaben, benzylparaben, and pentylparaben. Furthermore, hydroxybenzoic acid and its salts and esters—other than the esters mentioned above—are limited to a maximum concentration of 0.4% as acid for a single ester, and 0.8% for mixtures of esters [132].

Commission Regulation (EU) No. 1004/2014 inserted some changes in Annex V, which allows using butylparaben, propylparaben, sodium propylparaben, sodium butylparaben, potassium butylparaben, and potassium propylparaben at a maximum concentration of 0.14% (as acid) for the sum of the individual concentrations, and 0.8% (as acid) for mixtures of substances mentioned in entry 12 and 12a, where the sum of the individual concentrations of butyl- and propylparaben and their salts does not exceed 0.14%. However, in the same document, the use of these preservatives is prohibited in leave-on products designed for application on the diaper of children under three years of age [133].

#### 4.1.3. Aldehydes and Formaldehyde Releasers

Formaldehyde known as oxymethylene or formalin (37% concentrated solution of formaldehyde) is a preservative used in shampoos, shower gels, and liquid soaps. It is free or bound with formaldehyde releasers and it is not allowed in Japan [134]. Formaldehyde donors slowly release formaldehyde by degradation or decomposition under use conditions [135]. The antimicrobial activity of these preservatives probably results from formaldehyde released by hydrolysis in the presence of water [136]. Formaldehyde releasers are regulated on the basis of their formaldehyde release content [137]. A study carried out by Lv et al. [138] on eight formaldehyde-releasing preservatives, reported that formaldehyde release is dependent on the matrix, pH, storage time, and, above all, temperature. The positive list of Annex V includes: formaldehyde and paraformaldehyde, glutaral, imidazolidinyl urea, diazolidinyl urea, quaternium-15, DMDM hydantoin, bronopol, bronidox, hexetidine, and methenamine [4,139,140].

#### 4.1.4. Isothiazolinones

The isothiazolinone activity is related with the thiol and amine groups of their structures. These preservatives are often masked under the chemical names of their mixtures. Their usage is being diminished due to the large number of allergic reactions reported by dermatologists [141]. A study performed by Xia et al. [142] on quantitative structure-activity relationships (QSAR) of 22 3-isothiazolinone derivatives against *Escherichia coli*, showed that sulfur and nitrogen are the active sites of the molecule. Another study, carried out by Rezaee et al. [143] reported that three (2*H*)-isothiazolones substituted at the 5-position with chlorine are most lipophilic to those unsubstituted, and possess higher antifungal activity. Loss of chlorine can reduce antimicrobial activity. Additionally, an appreciable loss of activity is also noted in the presence of nucleophilic reagents (sulfhydryl groups), which suggests the possible elimination of chlorine by such groups [144].

Commission Regulation (EU) No. 1003/2014 stipulates that the use of the methylchloroisothiazolinone (and) methylisothiazolinone mixture is incompatible with the use of methylisothiazolinone alone in the same product because the 3:1 ratio allowed for the mixture would be modified [145]. On 22 July 2016, methylisothiazolinone was banned in leave-on products [146]. After 6 July 2017, the maximum authorized concentration of methylisothiazolinone was greatly reduced in rinse-off products (0.0015%) [147].

#### 4.1.5. Biguanides

The biguanides are a family of compounds known for their antimicrobial activities; they are used not only as antiseptics but also as preservatives [3]. Baker et al. [148] studied the structural determinants of the activity of some biguanides against the human oral flora. They revealed the following features: (1) alkyl chains can enhance antimicrobial activity over chlorophenyl groups; (2) the most lipophilic biguanides are the most active; (3) the antimicrobial activity increases as a function of the bridge length of the methylenes with a minimum bridge length of six carbon atoms; and (4) biguanides with terminal branches are more active than those with unbranched terminals. The biguanides allowed by the European Directive are chlorhexidine and polyaminopropyl biguanide [4].

#### 4.1.6. Quaternary Ammonium Compounds (QAC)

Quaternary ammonium compounds (QACs) mainly represent cationic surfactants. They are the most used antiseptics and disinfectants [149]. QACs may be considered as organically-substituted ammonium compounds, wherein the nitrogen atom has a valence of five; whereas four of the substituent radicals (R1 to R4) which are alkyl or heterocyclic radicals, and the fifth (X-) is a small anion. The antimicrobial activity of the QACs is a function of the length of the *N*-alkyl chain, which confers lipophilicity. Thus, for a QAC to have high microbicidal activity, at least one of the R groups must have a chain length in the C8 to C18 range [128]. The optimum activity against Gram-positive bacteria and yeast is obtained with chain lengths of 12 to 14 alkyls, while optimum activity against Gram-negative bacteria is obtained with chain lengths of 14–16 alkyls. Compounds with N-alkyl chain lengths <4 or >18 are virtually inactive [123,150].

The European directive Annex V, includes the following quaternary ammonium compounds: Alkyl (C12-22) trimethyl ammonium bromide and chloride (behentrimonium chloride, cetrimonium bromide, cetrimonium chloride, laurtrimonium bromide, laurtrimonium chloride, steartrimonium bromide, steartrimonium chloride), and benzalkonium chloride [4]. Regulation (EU) No. 866/2014 amended the use of cetrimonium chloride, steartrimonium chloride, and behentrimonium chloride at higher concentrations for rinse-off hair products, leave-on hair products, and leave-on face products [129].

#### 4.1.7. Nitrogen Compounds

Nitrogen is the most electronegative of all elements in Group V; this tends to impart a high degree of reactivity to the list of covalently bound nitrogen contributors. For discussion purposes, these can be divided into two groups: the first one corresponds to those that appear to react directly with a sensitive biological molecule, resulting in an inactive (or non-functional) end-product; and the second one is an adduct which combines with a sensitive site of the cell, resulting in the former inactivation [144].

Kabara et al. [151] performed a study about the relationship between chemical structure and antimicrobial activity of alkyl amides and amines. The authors concluded that: (1) Gram-positive bacteria are more sensitive than Gram-negative ones to the action of amines; (2) substituted amides of C8 to C12 are most active; (3) for *N*-amide to C18, addition of one epoxy group appears to contribute more to antimicrobial activity than unsaturation or halogenation. However, the addition of a second epoxy group does not improve this activity; (4) the lower alkyl amide of C12 is more active than those of a longer chain, and the addition of a second amide group at position 9 or 10 of the amide alkyl seems to increase antimicrobial activity.

Regarding the structures containing the pyridine moiety, these are excellent antimicrobials, due to the structural similarity with nicotinamide and pyridoxal [144]. Zinc pyrithione is a pyridine derivative and it was shown that the metallization of this compound greatly increased its biocidal action. Thus, the functional group *N*-hydroxythioamide of zinc pyrithione plays an important role in the molecular mechanisms of its biological action [152]. The electron withdrawing group, such as chlorine, improves the activity of isoxazole and pyridine. However, the electron-donating group, such as ethoxy, increases the strength of the compounds in the para position [153].

Considering the ethyl lauroyl arginate HCl, this compound was added to the positive list of preservatives in Annex V in 2013, its use being allowed to a maximum concentration of 0.4% (M1). Moreover, in 2016 a new amending done to Annex V allowed of the use of the ethyl lauroyl arginate HCl in mouthwashes (with restriction for children less than 10 years) [154].

#### 4.1.8. Heavy Metal Derivatives

Metal derivatives of mercury and silver are used as preservatives in cosmetics (thimerosal and phenylmercuric salts as organomercury compounds and silver chloride, according to Annex V) [4]. A central metal ion binds to the atoms of the donor ligands—such as O, N, and S—through often strong and selective interactions. Among the most important characteristics of metals is their ability to take part in redox reactions [155]. The heavy metals are toxic. They react with the proteins by complexing with the thiol groups (-SH), thus causing their inactivation [156].

#### 4.1.9. Inorganic Compounds

This class is represented by inorganic sulfites and bisulphites (Annex V). The most important factor that affects the antimicrobial activity of sulfites is pH. Sulfur dioxide and its associated salts exist as a pH-dependent mixture during aqueous dissolution [157,158].

### 4.2. Analytical Methods Used to Determine Preservatives

The protection of consumer’s health is the major concern of the institutional regulations, when the determination and establishment of the preservatives concentration limits are conducted. Despite the relatively high number of preservatives used in cosmetics, and the respective restrictions, there are a lack of formal analytical methods to control their presence in these products. In addition to the large number of substances to be monitored, the wide range of chemical structures and the variety of complex matrices present a major challenge for the development of reliable analytical methods [159].

Traditionally, the methods for the preservatives evaluation in cosmetics were mainly based on liquid chromatography with UV detection. Thin layer chromatography and electrophoretic methods have also been commonly used as separation techniques, in the development of identification and quantification methods [137,159]. The choice of the chromatography method is generally based on the physicochemical properties of the analytes. Liquid chromatography is chosen to determine the more polar and less volatile compounds, while gas chromatography is used to quantify the volatile components. Some study preservatives are derivatized using silylation or acylation reagents [160].

**Figure 4:**
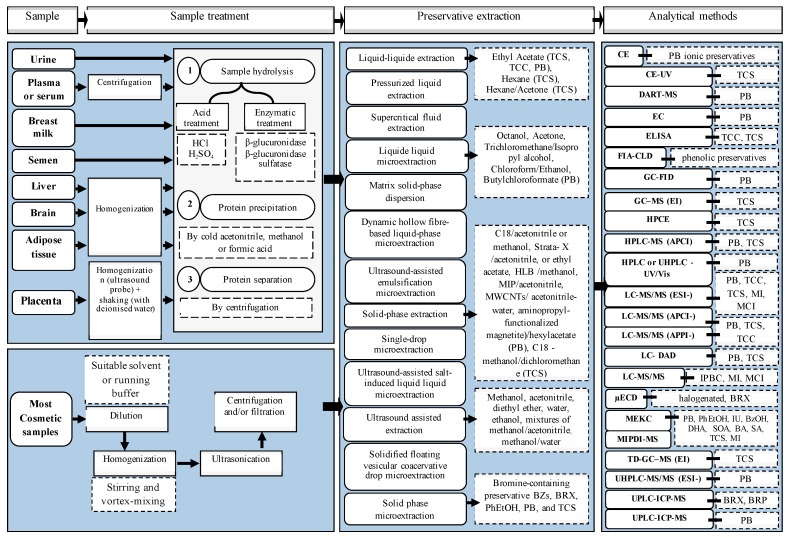
Steps followed in the analysis of cosmetic preservatives from the sample treatment to the analytical methods [137,159,160] where: µECD: microelectron capture detector; APCI: atmospheric pressure chemical ionization; APPI: atmospheric pressure photoionization; BA: benzoic acid; BRP: bronopol; BRX: bronidox; BzOH: benzyl alcohol; BZs: benzoates other than sodium benzoate; CE: capillary electrophoresis; CLD: chemiluminescent detection; DAD: photodiode array detection; DART: direct-analysis-in-real-time; DHA: dehydroacetic acid; EC (D): electrochemical (detector); EI: electron impact; ELISA: enzyme-linked immunosorbent assay; ESI: electrospray ionization; FIA: flow injection analysis; FID: flame-ionization detector; GC: gas chromatography; HLB: divinylbenzene/n-vinylpyrrolidone copolymer; HPCE: high-performance capillary electrophoresis; HPLC: high-performance liquid chromatography; ICP: inductively-coupled plasma; IPBC: iodopropynyl butylcarbamate; IU: imidazolidinyl urea; LC: liquid chromatography; MCI: methylchloroisothiazolinone; MEKC: micellar electrokinetic chromatography; MI: methylisothiazolinone; MIP: molecular imprinted polymer; MIPDI: microwave-induced plasma desorption ionization; MS: mass spectrometry; MWCNTs: multi-walled carbon nanotubes; PB: parabens; PhEtOH: phenoxyethanol; SA: salicylic acid; SOA: sorbic acid; TCC: triclocarban; TCS: triclosan; TD: thermal desorption; UHPLC: ultra-high performance liquid chromatography; UPLC: ultra-performance liquid chromatography; UV: ultraviolet; UV–VIS: ultraviolet–visible.

HPLC-based methods are still the most widely used in the literature for the analysis of more than one class of preservatives. In particular, methods based on reverse-phase liquid chromatography with columns C8 and C18 are the most commonly reported. Although UV detectors are the most popular ones, other detectors have also been used, such as mass spectrometry (MS), chemiluminescence (CL), electrochemical (EC), and so on [24,159,160]. The schema presented in Figure 4 summarizes the steps followed in the analysis of cosmetic preservatives from the sample treatment to the analytical methods.

### 4.3. Toxicity of Chemical Preservatives

The use of preservatives can induce undesirable effects for consumers, which can appear either after first contact or after years of cosmetic use. These effects range from mild irritation of the skin to estrogenic activity and, in the latest, it can be related with the mammary tumors inducing [137,161,162,163]. After perfumes, preservatives represent the second largest group of allergens most frequently implicated in cosmetic allergy [164]. There is a direct link between the antimicrobial effect and the ability to induce toxicity. This may explain why the most effective preservatives are often those with the greatest toxicity potential [165].

The European authorities have continuously updated the use of preservatives. The French National Agency of Medicine and Health Products Safety, has banned the manufacture, import, export, and marketing of cosmetic products containing chloroacetamide [166]. The Scientific Committee on Consumer Safety recommended new lower concentration limits for propylparaben and butylparaben, which it found to have “a low endocrine-modifying potential” [167]. On the other hand, triclosan was limited to a few cosmetic products at 0.3% and for mouthwashes at 0.2% [132]. In 2016, the use of methylisothiazolinone was banned [146], after a few months the European Commission published a new regulation limiting its use in rinse-off products to a maximum concentration of 0.0015% [147]. In June 2017, a draft Regulation was published by the European Commission which proposed to classify formaldehyde in Annex II (Prohibited Substances) of Regulation No. 1223/2009 on cosmetic products [4]. For this, the use of chemical preservatives as ingredients in finished products is subject to rigorous regulatory oversight in the different regions. The preservative safety test should include screening for acute toxicity, eye irritation, primary skin irritation, skin sensitivity, and basic mutagenicity test data. The sources of toxicity information for various preservatives are different, for example, the PCPC in the United States, which publishes safety reports known as the Cosmetic Ingredient Review (CIR) on the basis of independent scientific groups. Thus, Cosmetics Europe—The Personal Care Association is a similar professional association in Europe [168].

Typically, contact dermatitis (CD) is an eczematous reaction, usually to a substance applied to the surface of the skin. CD affects approximately 20% of the population in the United States [161,169]. Pathophysiologically, CD can be divided into allergic contact dermatitis reactions (affects 6% of the general population) and irritant contact dermatitis reactions [170,171].

When developing a new preservation system or selecting an existing preservation system for a cosmetic product, four main areas related to the assessment of consumer safety and risk assessment should be addressed: (1) hazard identification: potential toxic effects associated with a given material in preclinical and clinical assessments; (2) dose-response assessment: understanding the relationship between dose and effect incidence; (3) exposure: the actual use of the product by the consumer. In fact, the extent, duration, frequency and route of exposure can have a significant impact on the toxicity of a compound; and (4) risk characterization:placing the known hazards of an agent in the context of human exposure [168].

### 4.4. Selection of Appropriate Preservatives

Successful preservation depends on several factors that affect the antimicrobial efficacy and physicochemical stability of antimicrobial agents [30]. Overall, an ideal preservative should be stable, compatible, effective at low levels, non-toxic, consistent with cosmetic legislation, and non-expensive [137].

#### 4.4.1. Stability

Several factors may influence the stability of preservatives such as solubility and partition in oil/water (O/W) or water/oil (W/O) emulsions, formulation pH, and temperature during use, and the volatility of the preservative [8,100]. A good preservative must have a good O/W partition coefficient, since this will allow enhancing its activity in the aqueous phase of the formula [6]. In O/W emulsions, lipophilic preservatives, such as parabens, may be distributed in the lipid phase, and the product actually becomes unpreserved. Additionally, the distribution of preservatives in stacked products can compromise in situ efficiencies [30]. Thus, pH is an important parameter that can influence the stability of preservatives, either by provoking their decomposition or by modifying their conservative activity [5,6]. Parabens are, for example, ineffective in alkaline formulations due to their dissociation at this pH. Bronopol also undergoes slow decomposition at high pH. The effect of water on preservatives is very important. Formaldehyde donors may undergo slow decomposition in aqueous media. In contrast, the action of salts or alcohols depends on the osmotic effect [5].

#### 4.4.2. Compatibility

A suitable preservative must be compatible with the chemical compounds of a cosmetic formulation such as surfactants, solvents, dyes, perfumes, and other promotional additives [24]. In this regard, several preservatives will be inactivated by the antagonistic effect of certain cosmetic ingredients. Formaldehyde is influenced by many types of organic compounds, such as surfactants and nonionic proteins, and can lead to undesired side reactions in the formulation [5]. The antimicrobial activity of certain preservatives, such as parabens, may be altered, in particular, by non-ionic surfactants. On the other hand, the presence of high concentrations of solid minerals (carbonates and silicates, among others) or organic solids (cellulose and starch) causes absorption of preservatives. Talc, for example, decreases the antimicrobial activity of more than 90% of methylparaben [28]. In contrast, components, such as polyols and sunscreen active ingredients, can produce a synergistic effect with some preservatives [30]. EDTA is known for its synergy with several chemical preservatives; it disrupts the external lipid layer of bacteria and increases the penetration of other antimicrobial compounds into the cell [6].

Physical compatibility is also important. The addition of a preservative can influence the appearance of the cosmetic product and, for this reason, must be tasteless, odorless, and colorless [137]. The type of container used to package a cosmetic product will influence the concentration and activity of preservatives. Generally, lipophilic preservatives are associated with a greater risk of absorption by containers. Some containers are not compatible with certain preservatives, such as nylon with parabens or polyethylene with certain phenolic compounds, mercurial, and benzoates [28]. The influence of some cosmetic constituents on preservation is given in Table 2.

#### 4.4.3. Safety

A great part of preservatives have a low molecular weight, and thus can cause reactions of intolerance during the use of cosmetics. In general, the cosmetic industry has a major concern in finding effective and non-toxic substances [137,169]. Additionally, the safety factors and risks associated with the handling of antibacterial agents during manufacture must be considered [24].

However, sometimes the manufacturers do not respect the allowed concentrations of preservatives. Examples of these situations include the recovery of 24 cosmetic products because they contained methylisothiazolinone (0.025–0.36%), methyldibromo glutaronitrile, triclosan (0.4%), and benzalkonium chloride (1%), these concentrations being above the limits authorized by European Regulation 1223/2009. In another situation, 15 cosmetic products were recalled due to the presence of methyldibromo glutaronitrile, a preservative forbidden in cosmetics. Another product contained benzalkonium chloride at a concentration 10-fold higher than the maximum allowed. Moreover, 32 cosmetic products were recalled because they contained formaldehyde (0.3–25%) in concentrations above the established limits [2].

#### 4.4.4. Compliance with Cosmetic Legislation

The European Union and Japan regulate the use of the preservatives by a positive list published by official guidelines. In the European Union, the Annex V of the Regulation (EC) No. 1223/2009 of the European Parliament and of the Council of 30 November 2009, lists the authorized preservatives and their maximum concentration in ready for use preparation [4]. In Japan, Annex 3 of the “Standards for Cosmetics” of the Ministry of Health and Welfare (No. 331 of 2000) lists all preservatives authorized to be incorporated into cosmetics [134].

In the United States, there is no positive list of preservatives. The producer must take an autonomous responsibility for the safety of cosmetic products. The Cosmetic Ingredient Review (CIR) expert panel reviews and evaluates the safety of cosmetic ingredients. The CIR is an independent panel of industry-funded medical and scientific experts that meets quarterly to assess the safety of cosmetic ingredients based on the published literature, as well as others that are voluntarily funded by the cosmetic industry [173].

#### 4.4.5. Cost

The cost of cosmetic ingredients is a very important factor in their marketing. As a result, the industry still uses cheaper ingredients, rather than expensive ones [174]. The cost of cosmetics is influenced by several factors, including the cost of the raw material used, the costs of production, delivery, and marketing of the product. As a result, many cosmetics manufacturers and ingredient suppliers have turned to emerging markets such as ASEAN (Association of South East Asian Nations), Latin America, India, and China. The prices of products in these countries is relatively low, however, the increasing demand generate a growing price on the whole market. This has resulted in the need for many manufacturers to reduce their product prices in order to remain competitive. Customers in the ASEAN cosmetic industry are also able to choose low-cost alternative ingredients from local suppliers [175]. Now, the most important criteria that determine the selection of raw materials used are costs, market value, and availability. For example, several ingredients are used because of their availability and low cost, such as starch and many scleroproteins [176]. Overall, many consumers have shifted away from luxury brands to lower-quality products, including consumer and private-label products, particularly the “under-30” category [177]. The cost of active ingredients, such as antimicrobials, is not always a disadvantage on the marketing of the cosmetic product. A good example is handwashing with soap, in particular, which has been identified as the most cost-effective measure for disease control in various health promotion campaigns [178,179]. Studies have shown that hand washing could save more than a million lives annually from diarrheal diseases and respiratory infections, which are two of the leading causes of child mortality in developing countries. Even in developed countries, hand washing could prevent the spread of infectious viruses [178,180].

### 4.5. Preservative Mechanisms of Action

Unlike antibiotics, which act on specific sites of biosynthetic processes of microorganisms, preservatives act on multiple targets [104,181]. However, at sub-inhibitory concentrations, preservatives may act on a single target, what can lead to the development of resistance in microorganisms [182]. Preservatives can penetrate the cell envelope of Gram-negative bacteria by three routes: (1) the hydrophilic pathway, through porins; (2) the hydrophobic pathway by the lipid bilayer; and (3) self-promoting, which involves the displacement of divalent cations that bind adjacent lipopolysaccharide (LPS) molecules, thereby disrupting the structure of the outer membrane and exposing the phospholipid bilayer areas [182].

#### 4.5.1. Organic Acids

Organic acids have a broad antimicrobial spectrum. The individual activity of each acid varies according to several intrinsic or extrinsic factors, including pH variation [126]. Organic acids inhibit the growth of microorganisms by several mechanisms, including: (1) acidification of the external environment making it unfavorable to microbial growth; examples of acids used for this end are formic, acetic, propionic, butyric and benzoic acids [183]; (2) acidification of the cytoplasm by the penetration of uncharged organic acids into cells where the internal pH induces their dissociation into anions that consequently decreases the internal pH; this affects the isoelectric pH (pHi) of the functional enzymes involved in glycolysis, cell signaling and active transport, and proton-motor force (organic acids, e.g., propionic acid, benzoic acid, formic acids, sorbic acids) [126]; (3) changing the fluidity of the plasma membrane, this is typically achieved by medium- or long-chain organic acids (e.g., sorbic acids) [126,184]; (4) chelation and elimination of key nutritional trace elements or metal ions of the microbial shell by their complexation with negatively-charged anionic acids [183]; and (5) inhibition of enzymes from the cellular metabolism, such as the inhibition of fumarase, aspartase, and succinate dehydrogenase by sorbic acid, or inhibition of the active transport of some amino- and oxo acids by benzoic acid [126,182,183].

#### 4.5.2. Alcohols and Phenols

Alcohols and phenols are substances with effective antimicrobial properties. Their action is bactericidal, especially with acid-resistant bacilli. The mechanism of action of alcohol is related with the denaturation of proteins or inhibition of protein synthesis by several mechanisms [185]. Santos et al. [186] showed the impact of phenol-induced stress of *Pseudomonas putida* KT2440 on the relative abundance of proteins involved in the oxidative stress response, in the metabolism of lipids, amino acids, energy, nucleotides, and in division and cellular motility.

At low concentrations, benzyl alcohol and phenoxyethanol may induce membrane lysis in bacteria. Thus, they can denature the structure of proteins by binding to amino acid residues [187,188]. Phenoxyethanol also dissipates proton-motor force at low concentrations. *O*-phenylphenol inhibits the peptidoglycan biosynthesis by the inhibition of lysine biosynthesis in *S. aureus* [189]. At low concentrations, triclosan inhibits the enzymes of bacterial fatty acid biosynthesis (FabI or InhA (2-trans-enoyl-acyl carrier protein reductase) in *Mycobacterium* spp.) by forming a non-covalent complex with NAD^+^ of FabI [190]. However, at high concentrations, it induces a leakage of K^+^ leading to cell lysis by effects on RNA and protein synthesis [191,192]. In turn, the mechanisms of action of parabens are considered to be: (1) the inhibition of protein synthesis (including key enzymes, such as ATPases and phosphotransferases), by reacting with free amino acids, especially glutamic acid and aspartic acid [193]; (2) the inhibition of the synthesis of DNA and RNA [194]; (3) the influence on the transport of nutrients through the membrane [195]; (4) the interaction with mechanosensitive channels by allowing leakage of cytoplasmic contents [196]; and (5) the inhibition of oxygen consumption of mitochondria in fungi [197].

#### 4.5.3. Aldehydes and Formaldehyde Releasers

Aldehydes can react with chemical groups (amino, carboxy, thiol, hydroxyl, imino, and amide substituents) on biomolecules, including proteins and DNA. The crosslinking of proteins with formaldehyde leads to protein aggregation, resulting in irreversible chemical modification that leads to inhibition of metabolism and cell division [182,198].

Formaldehyde releasers act against bacterial cells by liberating formaldehyde in the medium. Despite this, the formaldehyde releasers can also react and undergo decomposition [199,200]. Generally, their biocidal effect is due to the proteins cell crosslinking, as well as RNA and DNA crosslinking. Kireche et al. [199] demonstrated that the reactivity of some formaldehyde releasers (DMDM hydantoin, bronopol, and methenamine) with amino acids and proteins is not related to the formaldehyde release. The antimicrobial activity of bronidox and bronopol is due to their oxidation of protein thiol causing inhibition of enzymatic activity and subsequent inhibition of microbial growth [201].

#### 4.5.4. Isothiazolinones

The isothiazolinones are oxidizing agents and their activity is due to their oxidizing effects on proteins, in particular on the thiol groups of the cysteine residues. This feature results in the inhibition of enzyme metabolism, as well as dysfunction of structural proteins in the cell wall and membrane [188].

#### 4.5.5. Biguanides

Among the biguanides, chlorhexidine is a positively-charged compound that binds to the negatively-charged membrane and bacterial wall resulting in significant damage. It promotes its own absorption so that it can reach its cellular targets. At low concentrations, it can lead to the loss of osmoregulatory and metabolic capacity, while, at very high concentrations, it can lead to a complete loss of membrane integrity and cause cytoplasmic coagulation [181,182].

#### 4.5.6. Quaternary Ammonium Compounds (QAC)

The QACs exert their antimicrobial activity by destabilizing the lipid bilayer of the plasma membrane of bacteria or yeasts and the outer membrane of Gram-negative bacilli, through association of the positive charge of quaternary nitrogen with the main polar groups of phospholipids (negatively-charged). The hydrophobic (alkyl chain) tail of the QACs acts later on the hydrophobic core of the membrane (the fatty acid chains) and destabilizes the interactions between the lipids and the membrane proteins [150]. The effects of QACs are based on their concentration, where: (1) at low concentrations, they induce a loss of osmogulatory capacity of the ions; (2) at intermediate concentrations, they disrupt membrane-associated systems such as respiration, solute transport and cell wall biosynthesis; and (3) at high concentrations, they solubilize the cell membrane components by forming micellar aggregates [123]. In summary, the antimicrobial activity of QACs mainly involves the rupture of membrane integrity and the leakage of cellular contents [202]. QACs can also denature structural proteins and enzymes by inducing ultrastructural changes [150]. Cetyltrimethylammonium bromide has an effect on DNA by binding to nucleic acids, provoking their precipitation [203].

#### 4.5.7. Nitrogen Compounds

Among the nitrogen compounds, zinc pyrithione has a broad spectrum of antibacterial and antifungal activities. Its mechanism of action consists of: (1) inhibition of transport through the membrane and membrane depolarization; (2) inhibition of the transmembrane proton motor force; and (3) acting as a metal complex [204,205].

Regarding triclocarban, this compound inhibits the growth of many Gram-positive bacteria, including MRSA and vancomycin-resistant Enterococcus, but it is not active against Gram-negative bacteria. However, fungi proved to be more resistant [128,206]. Triclocarban is an anilide that can act on the membrane by destroying its semi-permeable character. It also induces lysis of protoplasts in ammonium chloride by increasing the permeability to Cl [207].

In the case of piroctone olamine, is an antifungal compound with ability to reduce microbial colonization of *Malassezia* spp. [208]. It can penetrate the cell membrane and form complexes with iron (Fe^2+^ and Fe^3+^), by inhibiting energy metabolism in the mitochondria of target fungi [209].

#### 4.5.8. Heavy Metal Derivatives

Regarding the silver ions, these can cause: (1) inhibition of respiration by the interaction of silver with the thiol groups of the respiratory chain enzymes [210]; (2) membrane damage [211]; (3) reactive oxygen species (ROS) generation and interference with DNA replication [212]; and (4) the destruction of the proton motor force [213].

#### 4.5.9. Inorganic Compounds

Considering the inorganic compounds preservative mechanism, in particular sulfites derivatives, bacteria are the most sensitive. Additionally, sulfites are active against acetic acid bacteria, lactic acid bacteria, and Gram-negative enteric pathogens [214]. SO_2_·H_2_O diffuses passively through the microbial membrane [157]. The mechanisms of action of sulfites is related with: (1) reaction with cellular adenosine triphosphate (ATP) and/or (2) blocking of cystine disulfide bonds, leading to the inhibition of several cellular metabolism enzymes (including glycolysis) [158].

### 4.6. Microorganism’s Mechanisms of Resistance to Preservatives

Preservatives are used in cosmetics at low concentrations to minimize the risk of toxicity to consumers. However, this small quantity represents the major factor in the appearance of the resistance phenomenon in microorganisms. In addition, contamination rate, target type, temperature, environmental conditions, and contact time are other factors affecting microbial resistance. Preservative resistance may be considered as the inactivation of the preservative agent, the reduction in preservative efficacy, or a tolerance of microorganisms [215]. Generally, bacterial endospores (including *Bacillus* and *Clostridium*) are the most resistant forms. In contrast, mycobacteria (due to cell wall composition) are more resistant than Gram-negative bacteria being, however, Gram-positive bacteria most sensitive to preservatives [182].

Much research has been conducted to better understand the emergence of resistance to preservatives, recognized as a global problem limiting their use. Recent attention to current barriers and efforts on potential solutions, such as alternative models, are the basis for robust solutions. The development of new antimicrobials is crucial to fight resistance phenomena. Since there is a strong correlation between the use of preservatives and resistance development, alternative preservation forms, such as the ones based on emergent natural products, are necessary. In addition, establishing direct links between the fundamental axes of eco-evolutionary dynamics and the interactions between microbial species constitute future research needs, essential to tackle the problem of antimicrobial resistance.

#### 4.6.1. Organic Acids

The mechanisms of microorganism resistance to organic acids can be related to: (1) degradation of the organic acid; for example sorbic acid may be degraded to 1,3-pentadiene by some species of *Penicillium*, and benzoic acid is metabolized by several species of *Pseudomonas* and by *Acinetobacter calcoaceticus* [216]; (2) Adaptation of the microorganisms to the acid medium (the yeasts only adapt to small chain fatty acids), may be by using the H^+^-ATPase pump (i.e., proteins from the cell plasma membrane responsible by the molecules transport from or into cells; in this case, they transport the protons (H^+^) to maintain the pH), by the accumulation of the anions to buffer acid pH, or by the synthesis of acid shock proteins [183].

#### 4.6.2. Alcohols and Phenols

The most studied preservatives of this class are triclosan and parabens. Several mechanisms of microorganisms’ resistance to triclosan are the following: (1) modification of the target of triclosan (FabI) in *E. coli* [217]; (2) activation of the efflux pump (transmembrane proteins that provide active pumping, by consuming ATP energy, to evacuate unwanted molecules inside the cells. They operate by non-specific mechanisms in *E. coli* [218], *Salmonella enterica* serovar Typhimurium [219], *Acinetobacter baumannii* [220], *Campylobacter jejuni* [221], and *Stenotrophomonas maltophilia* [222]; and (3) swarming motility [223]. The microorganisms are resistant to parabens by: (1) enzymatic inactivation after hydrolysis to 4-hydroxybenzoic acid by esterase [224]; (2) superexpression of efflux pump genes [225]; and possibly (3) by porin deficiency [226].

#### 4.6.3. Aldehydes and Formaldehyde Releasers

Only two mechanisms of resistance have been revealed for formaldehyde: impermeability of cells and enzymatic inactivation. Mycobacteria can reduce the permeability of glutaraldehyde by changing monosaccharides of the arabinogalactan and arabinomannan fractions [227]. Thus, the permeability of glutaraldehyde can be reduced by the lipopolysaccharides of Gram-negative bacteria. Moreover, bacteria can resist formaldehyde via enzymatic degradation carried out by formaldehyde dehydrogenases [228].

#### 4.6.4. Biguanides

Lipopolysaccharides from Gram-negative bacteria represent a barrier to the permeability of chlorhexidine [229]. Efflux pumps are the most widely reported mechanism of chlorhexidine resistance [230]. The QACA protein (quaternary ammonium compounds A protein) is the most widely studied QAC effluent systems and it has been associated with an increased tolerance to chlorhexidine [181].

#### 4.6.5. Quaternary Ammoniums Compounds (QAC)

The external membrane and lipopolysaccharides of Gram-negative bacteria can be responsible for the high intrinsic resistance to QACs [182]. *P. aeruginosa* modifies the outer membrane ultrastructure by changing its fatty acid composition and phospholipids [231].

The mechanisms of resistance of microorganisms to QACs are different and it can be specified as follows: (1) reduction of the porins expression of the outer membrane (outer membrane proteins: OmpC, OmpF, and OmpA) [219]; (2) a mutational superexpression of the efflux pump genes, in particular, genes of QacA/B, QacC/D, Ebr, QacG, QacH, QacEΔ1, QacJ, multidrug efflux A (MdeA), norfloxacine A or B (ANorA, NorB), and multidrug export protein A (MepA) in *S. aureus*, acriflavine (AcrAB-TolC, AcrEF-TolC), YhiUV-TolC, EmrE, YdhE, MdfA, OqxAB, and TehA in *E. coli*, NorM in *Neisseria* spp., MdrL and Lde in *L. monocytogenes*, SdeXY in *Serratia marcescens*, or PmpM in *P. aeruginosa* [150,182,232]. The genes of these proteins can be expressed only for QACs or for other antimicrobial agents by cross-resistance [233].

#### 4.6.6. Heavy Metal Derivatives

Enzymatic inactivation is known as a mechanism of resistance in microorganisms by reduction to inactive metal. Organomercurial lyase (MerB) is an enzyme that cleaves the carbon-mercury bond in organomercurial compounds [234]. In addition, efflux pumps (e.g., MerE, MerC, and MerF) are another mechanism of resistance to organomercurials [213].

## 5. Conclusions

The antimicrobial efficacy is considered the main function of a cosmetic preservative. However, the inherent toxicity of these ingredients is a problem that the cosmetic industry should be concerned about. Therefore, it is necessary to continue the search for non-toxic and effective preservatives. The regulations limit, or even prohibit, the use of the most potent preservatives due to their toxicity and, in parallel, require uncontaminated cosmetic products. As a result, cosmetics manufacturers are seeking new preservation strategies to avoid regulatory requirements and, at the same time, to present a more secure product in terms of microbiological and toxicological aspects. On the other hand, a preservative has a restricted spectrum of activity depending on the target species and the forms of the microorganisms (spores, mycobacteria, Gram-negative bacteria, Gram-positive bacteria, yeasts, molds) which encourages manufacturers to use mixtures of them. In conclusion, cosmetic microbiologists face a great challenge looking for new alternative molecules by suitable criteria, new systems, or improved strategies of those already implemented.

## Figures and Tables

**Table 1:** Classification of cosmetic products with antimicrobial effects.

Class	Product	Application	Targeted Microorganism	Active Ingredient	References
Leave-on products	Deodorants	Inhibit the bacterial metabolism responsible for the degradation of sweat and subsequent production of unpleasant body odor	*Staphylococci* and diphtheroids of the *Corynebacteriaceae* family	Aluminum chlorohydrate, alcohol, triclosan, 3,4,4′-trichlorocarbanilide, chlorhexidine	[20,21]
	Antiperspirants	Suppress the release of sweat and eliminates the bacteria responsible for the unpleasant body odor production		Aluminum chlorohydrate, aluminum salts, zirconium-aluminum tetrachlorohydrex glycine complex	
Rinse-off hair products	Anti-dandruff shampoos	Reduces species of *Malassezia (Pityrosporum)*; Inhibit yeast growth and eradicate dead cells adhering to the scalp	The genus *Malassezia*	Zinc pyrithione, salicylic acid, imidazole derivatives, glycolic acid, steroids, coal, tar and sulfur derivatives, piroctone olamine	[20,21,22,23]
Skin care products	Antibacterial soap bars	Cleaning and bacterial reduction	*Staphylococci, Mocrococcus, Corynebacterium sp., Streptococcus*	Triclocarban, triclosan	[6,21,24,25]
	Disinfectants			Alcohol, triclosan, natural ingredients and glycerin	
	Antibacterial wipes			Benzalkonium chloride	
Face care products	Acne products and antiseptic cuticle treatment	Skin care; Cleaning and anti-acne treatments	*Staphylococcus aureus, Staphylococcus epidermis, Propionibacterium acnes*	Benzalkonium chloride	[8,21,24]
Oral care products	Toothpaste	Prevention of bacterial growth and plaque formation	*Firmicutes, Bacteroidetes* The families: *Proteobacteria, Actinobacteria, Spirochaetes, Fusobacteria* and the yeast *Candida albicans*	Triclosan, chlorhexidine, natural extracts	[21,24,26,27]
	Mouthwash			Alcohol+triclosan or alcohol+chlorhexidine	
	Antibacterial toothbrushes	Inhibit bacteria growth		Microban^®^, triclosan	

**Table 2:** Influence of some cosmetic constituents on preservation.

Component		Influence	Effects	Example	References
Solvent	Water	Negative	Main source of contamination	-	[20]
	Ethanol	Positive	Antimicrobial agent	Ethanol (more than 30%)	
Thickener and emulsifiers based on lipids		-	-	Fats, oils, waxes	
Surfactants	Cationic	Positive	Perturbation of cell membranes or increase in membrane porosity which also facilitates penetration of other antimicrobial substances	Alkylamines, quaternary ammonium compounds	[20]
	Anionic			Sulfates, sulfonates and carboxylates	
	Amphoteric			Alkylamidobetain and alkylamidoglycinate	
	Non-ionic			Fatty acids monoethanolamides, ethoxylated fatty alcohols and alkyl polyglucosides	
Humectants		Positive	At concentrations of 5 to 10%, they can effectively reduce the amount of biologically available water.	Sugars (sorbitol), glycerol and gylcol	[20]
Gelling agents		Positive	Antimicrobial agent and reduction of biologically available water	Polyacrylic acids and hydroxypropyl methylcellulose	[20,172]
Emollients		Negative	Promote the growth of microorganisms	Silicon derivatives, proteins (milk proteins and albumin hydrolyzate)	[20]
Plants extracts and mineral raw materials		Positive or negative	Positive: polyphenols can exert antibacterial effect; Negative: source of contamination especially for spores, mycotoxins and Clostridium	Melissa officinalis extract, rosmarinic acid and phenylethyl alcohol	[20,100]
